# Accelerated CMR using zonal, parallel and prior knowledge driven imaging methods

**DOI:** 10.1186/1532-429X-10-29

**Published:** 2008-06-05

**Authors:** Sebastian Kozerke, Sven Plein

**Affiliations:** 1 Institute for Biomedical Engineering, University of Zurich and Swiss Federal Institute of Technology, Zurich, Switzerland; 2 Academic Unit of Cardiovascular Medicine, University of Leeds, Leeds, UK

## Abstract

Accelerated imaging is highly relevant for many CMR applications as competing constraints with respect to spatiotemporal resolution and tolerable scan times are frequently posed. Three approaches, all involving data undersampling to increase scan efficiencies, are discussed in this review. Zonal imaging can be considered a niche but nevertheless has found application in coronary imaging and CMR flow measurements. Current work on parallel-transmit systems is expected to revive the interest in zonal imaging techniques. The second and main approach to speeding up CMR sequences has been parallel imaging. A wide range of CMR applications has benefited from parallel imaging with reduction factors of two to three routinely applied for functional assessment, perfusion, viability and coronary imaging. Large coil arrays, as are becoming increasingly available, are expected to support reduction factors greater than three to four in particular in combination with 3D imaging protocols. Despite these prospects, theoretical work has indicated fundamental limits of coil encoding at clinically available magnetic field strengths. In that respect, alternative approaches exploiting prior knowledge about the object being imaged as such or jointly with parallel imaging have attracted considerable attention. Five to eight-fold scan accelerations in cine and dynamic CMR applications have been reported and image quality has been found to be favorable relative to using parallel imaging alone.

With all acceleration techniques, careful consideration of the limits and the trade-off between acceleration and occurrence of artifacts that may arise if these limits are breached is required. In parallel imaging the spatially varying noise has to be considered when measuring contrast- and signal-to-noise ratios. Also, temporal fidelity in images reconstructed with prior knowledge driven methods has to be studied carefully.

## Introduction

The use of Magnetic Resonance (MR) methods in the cardiovascular realm continues to gain impact. A number of clinical indications have already been identified with Class I rating, including the assessment of global ventricular function and mass and the detection of acute and chronic myocardial infarction and myocardial scar [1]. A range of further applications have been indicated [2]. For example, recent results from a multi-vendor, multi-center clinical trial showed high diagnostic performance of myocardial perfusion MR imaging [3] compared with nuclear scintigraphy, suggesting that this method will have an important future role in clinical practice.

The technological development in cardiovascular MR (CMR) has been enormous with considerable innovations refining existing or even enabling new applications. A key driving factor for this innovation process has been the challenge to image in the presence of considerable object motion as it is found in the heart. Satisfying competing demands on total scan duration, image quality and spatiotemporal resolution has been a core motivation. With many CMR applications being performed during a breathhold, improving spatiotemporal resolution for a given, tolerable scan duration is of great importance. Improved spatiotemporal resolution allows discerning finer details of object structure and dynamics. Also, on a fundamental level, spatial and temporal resolutions determine image quality as the degree of partial volume artifacts, image fidelity of dynamic features and motion induced image distortions are affected.

In general, the relative discomfort due to lengthy breathholds, or in case of free-breathing acquisitions the overall scan duration, are considered drawbacks of CMR over other imaging modalities. In addition, the likelihood of image degradation due to irregular cardiac and respiratory motion tends to amplify with longer measurement times. Therefore, there has been high demand to improve the efficiency of data collection, thereby permitting improved spatiotemporal resolutions or reduced scan times or combinations thereof.

The relatively low imaging speed of MR is inherently linked to the sequential spatial encoding procedure by using time-varying magnetic field gradients. This principle leaves little freedom to reduce scan time for a given resolution and field-of-view in a conventional fashion due to constraints on the tolerable eddy currents induced in the body. A remedy to this problem is to reduce the number of encoding steps. This, inevitably, results in either reduced resolution if the density of the sampled points remains unchanged or in a reduced field-of-view if the density of sampled points is decreased. Since spatial resolution is not intended to be compromised, reduced scan time is typically traded for a reduced field-of-view. Because of the inverse relation between sampling density and field-of-view, one may refer to undersampling or reduced field-of-view methods collectively.

In some applications in which the object-of-interest is considerably smaller than the total field-of-view, scan time reduction by reducing the field-of-view and thus allowing foldover artifacts purposely is a straightforward approach and acceptable as long as the aliased portions do not fold on the object-of-interest itself. However, this strategy requires some experience in controlling foldover artifacts in particular with double-oblique scanning and only allows for very moderate reductions in scan time.

The concept of using a reduced field-of-view to shorten scan time can be refined by modifying the imaging pulse sequence such that signals from outside a desired reduced region-of-interest are suppressed or not excited [4]. This principle, which will be referred to as *zonal imaging *hereafter has been successfully used in a range of CMR applications [5-10].

On general grounds, however, suppression of magnetization cannot be ensured or achieved at all with any pulse sequence. Also, the amount of field-of-view reduction and thus scan time reduction is typically very limited given the extent of the object being investigated. To this end, treatment of foldover artifacts from a reduced field-of-view acquisition is required. In mathematical terms, an underdetermined problem is posed in such that a full field-of-view image is desired to be reconstructed from data acquired at a reduced field-of-view. In order to make this mathematical problem tractable, complementary spatial encoding functions are required. These are provided if the object signals are received with multiple, independent coils as proposed in first concepts in the late 1980s [11,12]. First practical implementations of *parallel imaging *using a k-space formalism [13] as well as the mathematical foundations from an image-domain perspective [14] followed ten years later. Today SMASH [13], its successor GRAPPA [15] and SENSE [14] are considered true milestones in MR technology with SENSE and GRAPPA being widely available on commercial MR systems. Instrumental to parallel imaging is the availability of dedicated receive coil arrays [16,17]. State-of-the-art systems permit the connection of up to 32-element coil arrays [18,19] with even more channels to become available in the near future. The quest for a larger number of independent channels is directly linked to expected improvements in parallel imaging performance with increasing numbers of channels. In addition, the extended coverage of many-element coil arrays can simplify patient setup as the actual positioning of the array on the patient becomes less critical. It has been shown that many-element coil arrays can be efficiently tailored to a specific target anatomy by deriving optimal virtual coil configurations [20].

Besides using parallel imaging principles, interesting opportunities for speeding up data acquisition arise in dynamic imaging. In dynamic imaging of the heart, the object-of-interest is embedded in static or only moderately dynamic structures such as the chest wall and the liver. In addition, individual, neighboring time frames of the heart are often very similar suggesting that considerable information redundancy is present in the data. A range of methods have been proposed attempting to exploit this information redundancy on its own or in conjunction with parallel imaging principles. The assumption of information redundancy being present can be regarded as prior knowledge and, accordingly, these methods are referred to as *prior knowledge driven*. Several methods have been proposed exploiting prior knowledge without and in combination with parallel imaging principles. Among those methods are UNFOLD [21], *k-t *BLAST/*k-t *SENSE [22], Compressed Sensing [23] and FOCUSS [24].

It is the purpose of the article to review the concepts and applications of CMR techniques that exploit zonal, parallel and prior knowledge driven imaging methods to speed up the imaging procedure. To give structure, the paper is subdivided into a concepts section addressing *zonal imaging*, *parallel imaging *and *prior knowledge driven *methods separately followed by an application section presenting preclinical or clinical examples of accelerated CMR imaging including a discussion of relative advantages and limitations of each approach.

## Concepts and implementation

**Figure 1:**
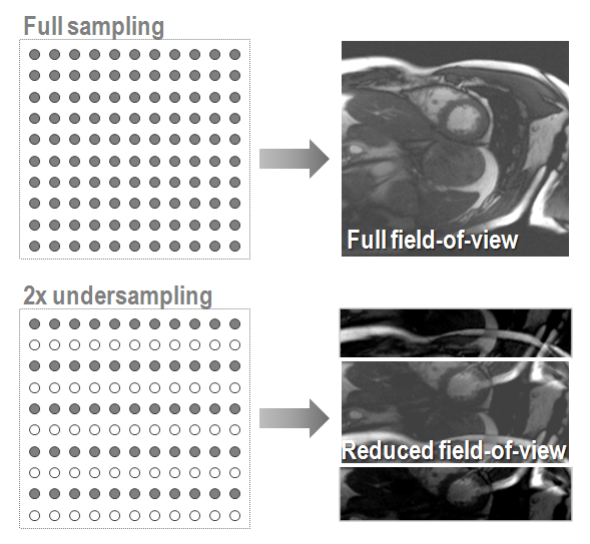
**Relation between sampling density and field-of-view. **Reduction of sampling density results in signals components folding into the desired, reduced field-of-view.

In the following a brief review of the underlying principles of undersampling techniques is given. For simplicity and for the fact that most clinically relevant CMR pulse sequences employ rectilinear sampling, only the Cartesian imaging case is considered herein. Accordingly, imaging scan time is assumed to be directly proportional to the number of phase-encode steps. If resolution is kept constant, scan time reduction by reducing the number of phase-encode steps straightforwardly implies a reduction of the field-of-view with resulting foldover artifacts. This is illustrated in Figure 1 for a short-axis slice of the heart reconstructed from 2-fold undersampled data.

### Zonal imaging

**Figure 2:**
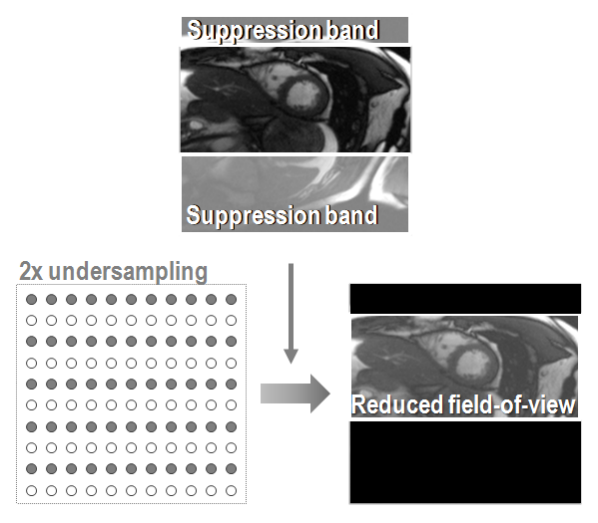
**Zonal imaging principle.** Signals from outside the desired field-of-view are suppressed using appropriate pulse sequence modifications. Accordingly, a foldover-free image is obtained.

In zonal or inner volume imaging, foldover artifacts from field-of-view reduction are removed by suppressing any signal from areas outside the reduced field-of-view as shown schematically in Figure 2. This signal suppression can be achieved by choosing perpendicular selection directions of two successive selective radio-frequency pulses in a spin-echo experiment [10]. Alternatively, two-dimensionally selective excitation pulses [5,6,8] or prepulses [9] as used with tagging [25] or strain-encoded acquisitions [26] can be used. These approaches are applicable to non spin-echo imaging modes. In general, the net speed gain from zonal imaging is inversely proportional to the extent of the object of interest relative to the full field-of-view. As a consequence, speed-up factors much greater than two cannot be achieved for common anatomy unless individual vessel cross-sections are to be imaged [27]. There are a few advantages of zonal imaging over other undersampling techniques. First, phased-array receive coils are not necessarily needed and image reconstruction involves the conventional Fourier transforms with its ease and speed. Second, suppressing unwanted signal contributions can be advantageous when using CMR motion tracking and correction techniques as signals from static regions do not interfere. Limitations of multi-dimensionally selective radio-frequency pulse are their relatively long duration and sensitivity to gradient imperfections and off-resonances. Parts of those problems can be alleviated by reducing the pulse duration in combination with the transmit analogue of parallel imaging [28], an area which sees significant research efforts in view of tackling eminent problems at very high magnetic field strengths.

### Parallel imaging

**Figure 3:**
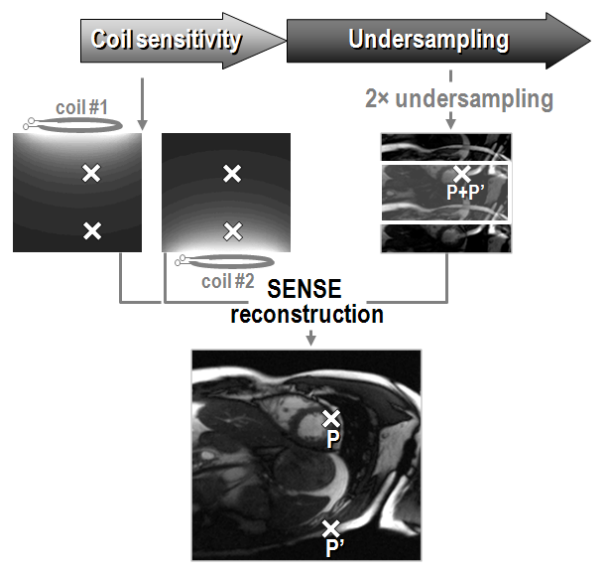
**Parallel imaging principle.** A reduced field-of-view acquisition results in image foldover (for example, point P contains signal from two points (P and P') in the original). The folded image pixels are unfolded using differences in coil sensitivity of the two antenna elements (coil #1 and #2).

The principle of parallel imaging is illustrated in Figure 3. In this exemplary illustration, two-fold undersampling results in two signals overlapping in each point of the image, if reconstructed conventionally. For example, the image intensity seen at point P as indicated in Figure 3 is the superposition of the original signal at that point and the signal folded in from position P' located at position P plus the field-of-view divided by two (= the reduction factor). In order to unfold the signal received in point P additional information is required since two unknowns exist with only their superposition being known. This additional information is derived from the difference in signal reception in the receive coils placed around the object. In the example, two receive coils having different sensitivities with respect to point P and point P' are required. By calculating the difference in sensitivity of coil #1 and coil #2 for all image points, all folded image pixels can be reassigned successively.

The ability to reassign folded signal intensities depends on how many signals actually fold on top of each other. It also depends on the orthogonality of the coil sensitivity functions and thus the coil array configuration and the wave length of the radio-frequency fields involved. The latter is determined by the static magnetic field strength. In the range of clinically available magnets for CMR ranging from 0.5 to 3.0 Tesla, wave length effects play only a minor role. Accordingly, the performance of parallel imaging is primarily determined by the speed-up or reduction factor and the number of coil elements and their configuration with respect to the object being imaged.

#### Signal-to-noise considerations

**Figure 4:**
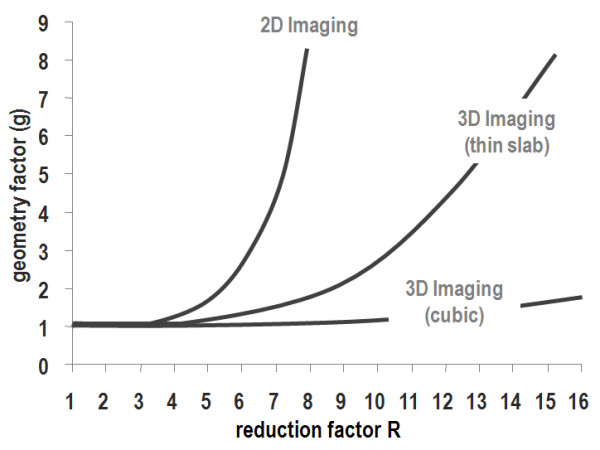
**Noise amplification in parallel imaging. **The additional noise penalty as expressed by the geometry factor is shown. For 2D imaging the critical reduction factor is between 3–4. In 3D imaging with comparable dimensions and reduction factors along the two phase-encode axes the critical reduction factor is about 16.

In general, and this applies to all undersampling methods, reducing the number of sampled data points reduces the signal-to-noise ratio or, if assuming unit signal response, increases the noise in the reconstructed images by the square-root of the reduction factor. Beyond this obvious penalty, noise can further be amplified in parallel imaging due to nonorthogonality of the coil encoding functions as shown in Figure 4 for increasing reduction factors and different imaging modes. This additional noise penalty is reflected by the geometry or g-factor [14] and leads to spatially varying noise across the image. In theoretical work it has been shown that the g-factor relation is governed by fundamental electrodynamics [29,30]. In the 2D Cartesian imaging situation, undersampling can only be applied along one phase-encode direction. The critical reduction factor is defined as the cut-off beyond which image degradation due to noise increases exponentially. According to Figure 4, the critical factor for 2D imaging is around 3 to 4. It is emphasized again, that the g-factor does not reflect the inherent noise amplification due to scan time reduction; instead it gives the additional noise penalty specific to parallel imaging. In the 3D imaging case, in which undersampling along two dimensions becomes possible [31] the critical limit can be extended to 16, with great prospects for highly accelerated volumetric 3D imaging [19]. Obviously, a prerequisite of applying these large reduction factors is sufficient base signal-to-noise, since 16-fold scan time reduction alone accounts already for 4-fold decrease in the signal-to-noise ratio.

#### Implementations

On today's systems, SENSE [14] and GRAPPA [15] are most widely available. The two methods differ in terms of the domain in which the reconstruction problem is solved and with respect to how coil sensitivities are derived. As the image and k-space domains are linked linearly, both SENSE and GRAPPA reconstructions can be formulated using a common mathematical framework [32]. Using this framework it can be shown that GRAPPA is an approximation of SENSE. A real difference between SENSE and GRAPPA implementations refers to the derivation of coil sensitivities. While in most SENSE implementations coil sensitivities are obtained from a separate low-resolution volumetric reference scan, GRAPPA acquires a few additional profiles encoding the full field-of-view during actual data acquisition for coil calibration. The latter is often referred to as auto-calibration [33]. Auto-calibration has the advantage of ensuring consistency between coil sensitivities and undersampled data if motion during or in-between successive scans occurs. The downside of this approach is a reduction of the net acceleration factor of the actual acquisition which is smaller than the nominal undersampling factor as reported frequently in the literature.

In dynamic imaging application the undersampling pattern can be shifted as a function of time, such that coil sensitivities can be estimated from a time-average image. Thereby additional calibration data are not required. The use of interleaved sampling for calibration purposes in combination with SENSE reconstruction is known as TSENSE [34]. In UNFOLD-SENSE [35], the time-interleaving and with it the displacement of static and dynamic signal distributions in the field-of-view along the temporal frequency axis is exploited in combination with a temporal filter to reduce residual artifact from parallel image reconstruction.

### Prior knowledge driven imaging

**Figure 5:**
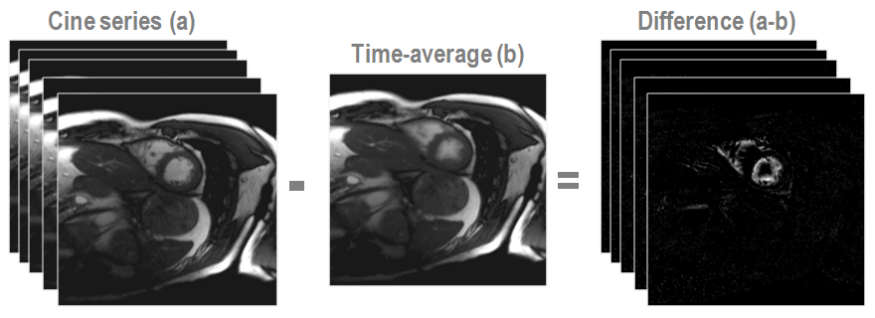
**Information redundancy.** In this example the time-average of all cine image frames is subtracted from each original image frame to illustrate the sparseness of information contained in the cine series. It is seen that the difference images contains only very few image pixels with significant values.

Prior knowledge driven methods are used to exploit information redundancy in dynamic CMR image series. The amount of redundant information present in a cine series of the heart can be visualized using a simple experiment. If one subtracts the time-average over image points from each individual image frame of the series, only the differences between the average and each individual frame remain (Figure 5). Those difference maps represent the image in a much sparser fashion with only very few significant signals. Based on this observation, undersampling and reconstruction strategies have been developed to accelerate the acquisition of objects presenting with large degrees of image correlation in space and time such as the heart embedded in the chest.

Several strategies have emerged using image correlations as such [21,22,24,36,37] or in combination with parallel imaging [22,24,35,38]. Widely available on current systems are *k-t *BLAST and *k-t *SENSE [22]. These methods also use a time-interleaved, shifted undersampling similar to the TSENSE and UNFOLD-SENSE methods. Besides sequentially shifted undersampling, optimal undersampling pattern can be derived [39]. Like in TSENSE, auto-calibration for parallel imaging from a time-average image is applicable.

**Figure 6:**
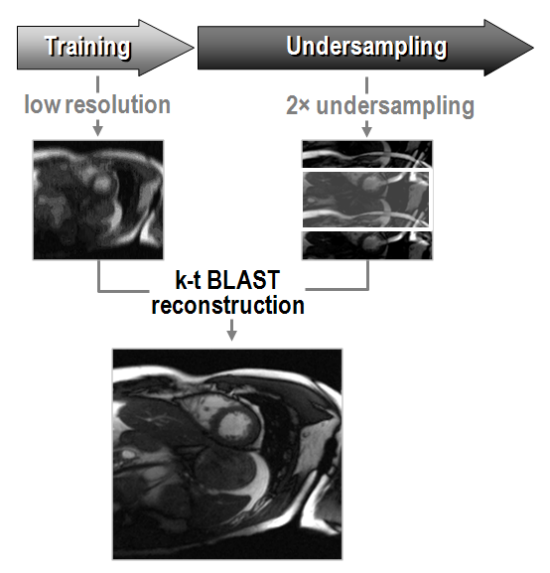
***k-t *****BLAST principle.** Based on low-resolution training data, an estimate of the expected signal intensities of the object is obtained and used to unfold the aliased components resulting from undersampled data acquisition.

In *k-t *BLAST and *k-t *SENSE an image estimate is obtained in a so-called training stage. In this training stage, images are acquired at very low spatial but at full temporal resolution. These data may either be obtained in a separate scan or in an interleaved fashion during the actual acquisition [40,41]. During the undersampling stage, sparse sampling is applied along k-space and time (k-t imaging). To obtain a high-resolution, unaliased image series, the prior knowledge as derived from the training data is used to unfold the image as outlined in Figure 6. In parlance of image reconstruction, the training data determines a filter which suppresses folding artifacts as a result of undersampling. While this reconstruction filter is the only handle to resolve foldover in *k-t *BLAST, coil sensitivities are used in addition in *k-t *SENSE.

**Figure 7:**
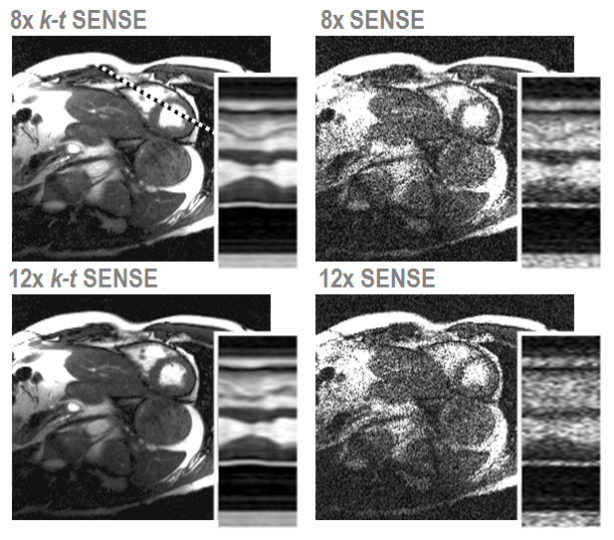
**Comparison of *k-t *SENSE and SENSE at high acceleration using a 32-element coil array.** Short axis views obtained from 2D imaging at nominal reduction factors of 8 and 12 are compared (net reduction factors: 5.6 and 7.3). Spatiotemporal plots were taken along the dashed line indicated.

In general, *k-t *SENSE tends to outperform its frame-by-frame SENSE counterpart for comparable reduction factors (Figure 7), even if state-of-the-art 32-channel coil arrays are employed. It is, however, to be noted that *k-t *SENSE is only applicable to cine or dynamic applications, while parallel imaging applies to all kinds of acquisition strategies.

**Figure 8:**
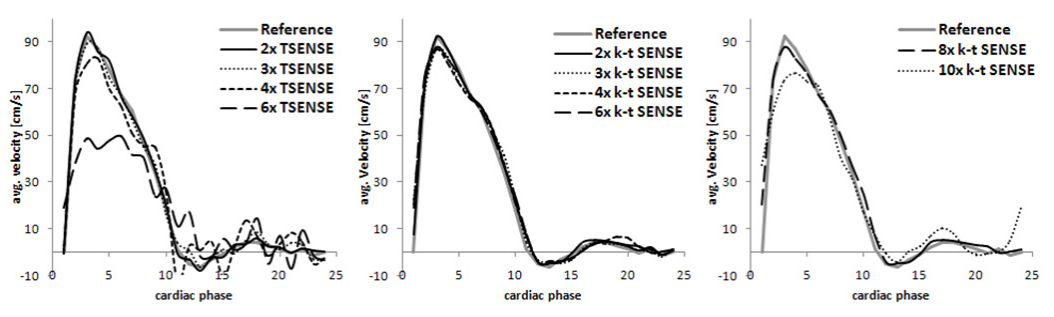
**Comparison of TSENSE and *k-t *SENSE reconstructions of data decimated to different reduction factors from a fully sampled phase-contrast flow measurement acquired with a 32-channel coil array.** Aortic velocity data as a function of cardiac phase from TSENSE and *k-t *SENSE with net reduction factors of 2, 3, 4 and 6 are compared relative to values obtained from the fully sampled reference (left, middle). Training data plug-in is used in all *k-t *SENSE reconstructions. Significant temporal filtering is seen for net reduction beyond factor 8 with *k-t *SENSE (right).

Limitations of *k-t *BLAST and *k-t *SENSE can include insufficient un-mixing of different temporal frequency components folded on top of each other which can lead to temporal blurring and flickering in the data. To alleviate the effect of temporal blurring at high reduction factors, training data plug-in has been proposed [42]. Thereby training data are substituted into the final k-space reconstruction ensuring data consistency. A comparison of blood velocities obtained in the ascending aorta with up to 8× net reduction using *k-t *SENSE with training data plug-in relative to SENSE/TSENSE reconstructions is shown in Figure 8. Superior noise suppression with *k-t *methods at high reduction factors is traded for pronounced temporal filtering as exemplified for 10× net reduction. Temporal filtering seen at at high reduction factors can be reduced by using an increased number of training data profiles [43] which, however, offsets the initial speed gain. As an alternative, parallel imaging can be employed to boost the training data resolution without time penalty. To this end, however, careful considerations as to the minimum acceptable SNR in the training data are required. The low resolution in the training data also demands dedicated SENSE reconstruction methods to avoid residual folding artifacts [44].

#### Signal-to-noise considerations

As for parallel imaging, image noise is non-uniform across the image with k-t methods. In parallel imaging, this non-uniformity is directly accessible from g-factor maps and depends on the reduction factor and coil array configuration. In k-t methods, the noise also depends on object dynamics. For example, in static or slowly moving structures, the noise variance will be much smaller compared to areas with highly dynamic features. This noise non-uniformity found with parallel imaging and k-t methods has implications for practical signal-to-noise ratio measurements. To this end methods have been proposed [45,46]. However, difference methods [45] may render inaccurate results in particular in cardiac imaging, because truly identical object states are difficult to capture with successive images. Instead of using a difference method it is proposed to repeat the sequence with all radio-frequency pulse switched off or obtain noise only data during a short prescan [46]. This does not require respiratory control or breathholding as spatial information is not encoded and would provide together with the signal data true signal-to-noise ratio assessment. Such a scheme is currently offered by at least one major vendor.

### Accelerations achievable with parallel and prior knowledge driven CMR

A number of parallel imaging and prior knowledge driven methods as well as hybrid methods of both concepts have been proposed and applied to CMR. In Table 1 examples of these approaches are given with references for their practical CMR application as well as nominal and net reduction factors reported. While nominal reduction factors specify the amount of undersampling without taking into account data sampled to populate auto-calibration or training data, net reduction factors denote effective reduction factors. Unfortunately, net reduction factors are not always reported in the literature and actual scanner implementations may differ from what is described in publications. Accordingly, for Table 1, assumptions had to be made to estimate net reduction factors. For methods with external calibration, nominal and net reduction factors are assumed to be identical.

**Table 1:** 

**Parallel Imaging**	**Prior Knowledge Driven Imaging**	**Hybrids**	**Prior Knowledge**	**CMR Applications**	**R_nom_/R_net_**	**References**
SENSE			-	cine, rt, dce, lge, qflow, cmra	1.5–8.0	[19,55,64,66-68,73,78,82-84]
SMASH			-	cine, cmra	2.0	[16,85-87]
GRAPPA			-	cine, cmra	2.0/1.6	[15,53,88]

	UNFOLD		constant x-f support	cine, rt, dce	2.0	[21,89-92]
	k-t BLAST		Measured x-f support	cine, rt, dce, qflow	5.0–8.0/4.0–5.7	[22,39,40,42,51,57,69,70,93]
	CS		sparse x-f support	cine, qflow	2.0–7.0	[23,24,94]
	TCR		temporal constraint	dce	4.0–5.0	[37]

		TSENSE	temporal correlation^1)^	cine, rt, dce, dense	2.0–7.0	[34,52,65,75,91,95-98]
		TGRAPPA	temporal correlation^2)^	cine	2.0–4.0	[99]
		k-t GRAPPA	temporal correlation^3)^	cine, cmra	3.0–7.0/2.0–5.2	[38,100 ]
		k-t SENSE	Measured x-f support	cine, rt, dce, qflow	5.0–8.0/4.0–5.7	[22,39,41,42]
		FOCUSS	measured, sparse x-f support	cine	4.0–16.0	[24]
		UNFOLD-SENSE	constant x-f support	cine, dce	2.0–3.0/1.8–2.7	[35]

^1)^coil sensitivity weights from temporal average of data sampled on a sheared grid (cmp. DC signal in x-f support)

^2)^auto-calibration signals (acs) from temporal average of data sampled on a sheared grid

^3)^with or without acs lines from temporal average of data sampled on a sheared grid

x: spatial position, f: temporal frequency

cine: cardiac-gated cine imaging; rt: non-gated real-time imaging; dce: dynamic contrast-enhanced imaging; lge: late gadolinium enhancement; qflow: quantitative flow imaging; cmra: coronary magnetic resonance angiography; dense: displacement-encoded imaging

R_nom_: nominal reduction factor; R_net_: net reduction factor taking into account calibration or training data (only reduction factors used in 2D CMR applications are reported here; in case net reduction factors are not available from literature a matrix of 200 in phase-encode direction and 11 auto-calibration or training profiles acquired interleaved are assumed).

Critical reduction factors beyond which the diagnostic value of reconstructed images is compromised have been indicated and are largely in agreement with theoretical considerations and experimental g-factor measurements for parallel imaging methods [29,30]. In 2D imaging applications using SENSE, TSENSE, GRAPPA, TGRAPPA this critical factor amounts to about four. In 3D imaging with large coil arrays, greater reduction factors have been achieved [47,48] but the critical value with respect to diagnostic image quality achievable in CMR applications remains to be determined. Using prior knowledge driven methods in combination with parallel imaging, net reduction factors up to 16 have been realized [24]. The diagnostic value of images acquired at such high reduction factors remains, however, to be shown. CMR studies using *k-t *BLAST and *k-t *SENSE have indicated temporal filtering effects for net reduction factors greater than eight compromising the true temporal resolution in dynamic data.

## Clinical application

Parallel imaging and k-t methods that are available on clinical scanners, such as SENSE, GRAPPA, TSENSE, *k-t *SENSE and *k-t *BLAST, provide operators with flexible and important tools to optimize a range of CMR protocols. The increased scanning efficiency afforded by these methods can be used to shorten imaging times especially in patients with poor breathhold capacity with resulting improvements in image quality. Alternatively, spatial and temporal resolution as well as coverage can be increased.

### Cine imaging of cardiac function

**Figure 9:**
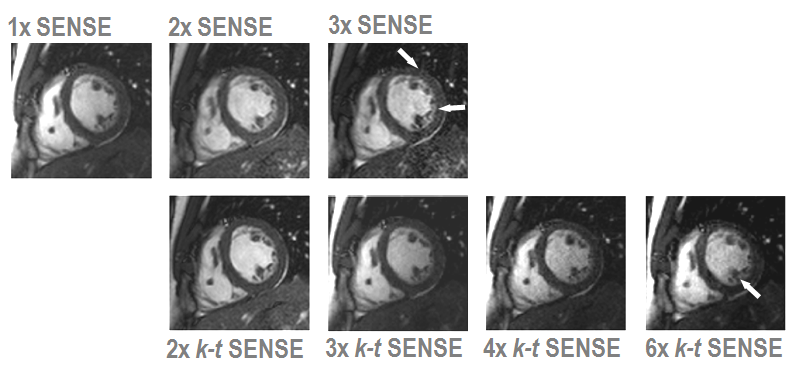
**Comparison of clinical 2D cine imaging in the short axis orientation acquired with SENSE and *k-t *SENSE using a 6-element coil array.** Each image depicts the end-diastolic frame of separate breath-hold cines acquired with 1× to 3× SENSE and 2× to 6× *k-t *SENSE. 2× SENSE provides excellent image quality. With 3× SENSE, the SNR reduction and image artifacts become apparent (arrows). *k-t *SENSE up to 4-fold net acceleration results in good image quality. At 6× *k-t *SENSE, slight blurring of the endocardial wall and papillary muscles is visible (arrow).

Cine imaging is an essential component of virtually every CMR study. Measurements of regional and global ventricular function are typically derived from a stack of multiple 2D cines. Depending on the clinical application, further cine series in customized imaging planes are often performed. With current balanced steady-state free-precession pulse sequences the acquisition time for each 2D slice is on the order of 10 seconds to achieve acceptable spatial and temporal resolution [49]. Accordingly, cine imaging accounts for a large proportion of the scan time in most CMR studies. Therefore, parallel imaging methods with two to three-fold acceleration are often used in clinical cine protocols (Figure 9) reducing the scan time per slice to about 4–5 seconds. As expected from theory, beyond three-fold acceleration, SNR limitations increasingly affect the clinical utility of the acquired data. Several studies have shown that cine imaging with SENSE and other parallel imaging methods yields comparable measurements of left and right ventricular volumes and function as conventional cine imaging [50].

Further acceleration can be achieved with k-t methods reducing acquisition times for a 2D cine scan to 2–3 seconds per slice. As seen in Figure 9, image quality is better preserved at higher acceleration than with parallel imaging alone. Initial studies have suggested that 2D cine imaging with *k-t *BLAST [51] and TSENSE [52] provide similar measurements of cardiac function as non-accelerated acquisition as long as the net acceleration factor does not exceed four to five. For larger reduction factors, underestimation of ejection fraction has been noted [52].

**Figure 10:**
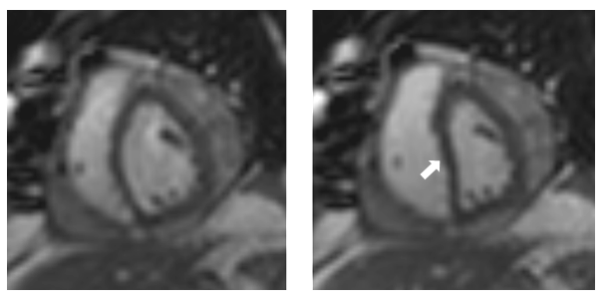
**Real-time imaging with 2× SENSE in the short axis orientation in a patient with recent mitral valve replacement surgery.** Images captured during deep expiration (left) and inspiration (right) show diastolic inversion of the interventricular septum in inspiration consistent with constrictive pericardial disease.

Parallel imaging methods also allow for so called "real-time" cine imaging, in which multi-phase data sets are acquired with a continuous dynamic acquisition [53]. While such methods have not yet found a role for the assessment of cardiac function and dimensions, potential applications of 2D "real-time" cine imaging include for example the study of the motion of the interventricular septum in suspected constrictive pericardial disease [54] (Figure 10).

A different use of increased scan efficiency is time-resolved 3D imaging. In particular in combination with many-element coil arrays, parallel imaging can provide sufficient acceleration to image the entire heart within a single breathhold at spatial and temporal resolutions comparable to conventional 2D cine methods [55]. Prior knowledge driven techniques can also be employed to facilitate single-breathhold 3D cine imaging [40,51]. The clinical role of 3D cine imaging is yet to be determined, but its potential benefits are easier planning, faster data acquisition and avoidance of misregistration that can affect 2D imaging with multiple breathholds. For volumetric measurements, initial studies have suggested a small systematic underestimation in particular of the end-diastolic volume in 3D compared with 2D cine imaging, which may be related to the effects of misregistration or to the encoding process [51,56].

Three-dimensional cine imaging has also been successfully applied to dobutamine-stress MRI, where cardiac function has to be assessed repeatedly and rapidly at different stages of pharmacological stress. Segmental wall motion analysis from *k-t *BLAST 3D cine imaging was similar to conventional cine SSFP imaging at rest and during dobutamine stress [57].

### Real-time imaging

Besides its use in interventional applications [58-60], advanced acquisition schemes combined with rapid reconstruction also permit the acquisition of CMR data in real-time [61-63]. Real-time methods are of great importance for imaging patients with irregular sinus rhythm and poor breathhold capability. There has been a number of studies indicating the benefits of improved spatiotemporal resolution by using parallel imaging methods for assessing functional indices [53,64,65], diagnosing wall motion abnormalities [66] or quantifying blood flow velocities [67] using real-time methods.

### Velocity-encoded imaging

Accelerated quantitative flow imaging has been of interest in a range of applications. Given the small diameter of blood vessel, zonal imaging techniques have been used to facilitate real-time flow quantification during hyperemic response [7,27]. Flow imaging accelerated with parallel imaging techniques has been used for shunt quantification in children [68]. The speed gain with *k-t *BLAST has also been demonstrated to reduce very long scan time associated with velocity spectrum mapping or Fourier velocity imaging [69]. Application for velocity quantification in stenotic valves has been described [70]. Quantitative flow data obtained with *k-t *SENSE have been shown to agree well with the reference up to reduction factors of eight [42]. Beyond net reduction factors of 8 temporal filtering of blood velocity curves is noticeable in accordance to the data presented in Figure 8.

### Coronary and anatomical imaging

The reliable non-invasive visualization of the coronary arterial system remains one of the most rewarding prospects of tomographic imaging. Although coronary computed tomography currently provides superior image quality, CMR coronary angiography remains an important alternative because it does not require the use of contrast agents and does not expose patients to ionizing radiation. Current clinical applications for coronary MRI are the assessment of anomalous coronary arteries and other congenital pathologies [1]. Imaging of the coronary venous system for the planning of electrophysiological studies is emerging as a potential future application.

**Figure 11:**
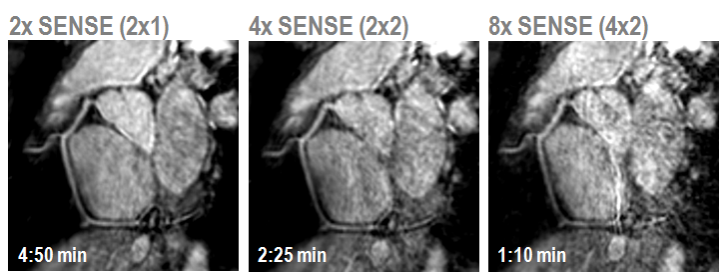
**Free-breathing, whole-heart coronary MRA with SENSE using a 32-element coil array. **The path of the right coronary artery has been tracked. Up to 4-fold acceleration SNR losses are acceptable. At 8-fold acceleration noise enhancement compromises diagnostic quality.

Accelerated data acquisition is essential to permit the acquisition of a 3D imaging slab that covers the entire heart with the required spatial resolution and within an acceptable time frame. So called "whole-heart" CMR with typically 90 to 120 slices and a spatial resolution of approximately 1 × 1 × 1 mm^3 ^is usually acquired with the help of respiratory navigators [71]. Parallel imaging is used in most whole-heart protocols in order to keep imaging times below 10 minutes. Further acceleration is feasible, especially with larger coil arrays [19]. As with all applications of parallel imaging, SNR and image quality are offset against the advantage of shorter acquisition time as demonstrated in Figure 11.

The same "whole-heart" imaging methods developed for coronary imaging can be employed for anatomical assessment of other cardiac structures. The high spatial resolution of the acquired 3D data sets provides a useful basis for assessment of congenital heart disease and other morphological studies [72].

### Dynamic imaging of myocardial perfusion

**Figure 12:**
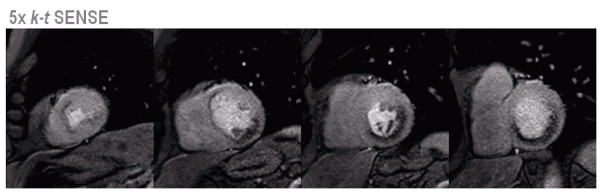
**Adenosine stress myocardial perfusion imaging with an acquired in-plane spatial resolution of 1.3 × 1.3 mm^2 ^facilitated by 5× *k-t *SENSE. **Four short axis slices are shown covering the heart from the apex (left) to the base (right). A large infero-lateral perfusion defect and anteroseptal ischemia in the apex is seen. Due to the very high spatial resolution, endocardial dark rim artifacts are minimal and the spatial extent of the perfusion defects is clearly delineated.

The principal challenges of dynamic first pass myocardial perfusion imaging are to acquire data with high temporal resolution to suppress motion artifacts, to provide sufficient spatial coverage to delineate ischemia in all coronary segments and to deliver sufficient spatial resolution to minimize imaging artifacts such as endocardial dark rims. In order to satisfy all of the requirements, gradient echo perfusion CMR has been combined with echo planar imaging and SENSE [73,74] or TSENSE [75]. With these methods, three to four imaging sections can typically be acquired at every heart beat in an acquisition time of around 100 ms per slice and an in-plane spatial resolution of 2–3 mm [73,74]. Recently, prior-knowledge driven myocardial perfusion imaging using *k-t *BLAST and *k-t *SENSE has been proposed. The additional acceleration has been invested in either a shorter acquisition time per slice [57] or improved spatial resolution [41]. In particular the latter approach appears to be promising, as imaging artifacts such as endocardial dark rims, were reduced in line with the spatial resolution and the ability to assess transmural perfusion distribution was enhanced (Figure 12).

### Imaging of myocardial viability

**Figure 13:**
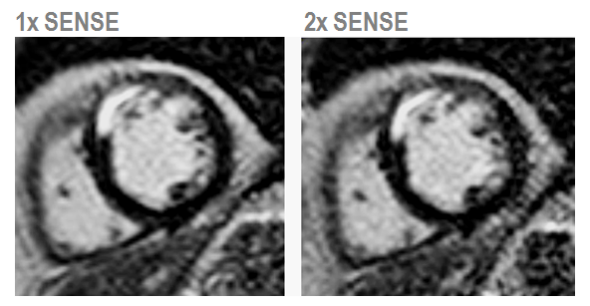
**Comparison of 2D late-contrast enhanced MR imaging without and with 2× SENSE acceleration and using a 6-element coil array. **Both images provide similar image quality with clear delineation of an antero-septal scar. The SNR decrease is seen with 2× SENSE, best appreciated in the lateral wall.

Late gadolinium-enhanced (LGE) CMR for the assessment of myocardial viability has become a cornerstone in the diagnosis of ischemic and non-ischemic cardiomyopathies [76]. The method provides unparalleled delineation of fibrotic or destructive processes in the myocardium with high contrast to normal tissue. However, each section of a 2D stack of late contrast-enhanced data has an acquisition time of 10–12 seconds [76], so that coverage of the whole heart can require up to 10 minutes of scan time. Because of the typically high contrast between normal myocardium and scar, LGE is well suited for acceleration with parallel imaging methods with acceleration factors of 2 and 3 in 2D and 3D imaging protocols [77]. As can be seen in Figure 13, image quality and diagnostic yield are comparable between non-accelerated and 2× SENSE imaging. Image-based motion correction techniques in combination with single-shot SENSE have been developed to improve signal- and contrast-to-noise ratios in LGE by averaging [78].

## Conclusion

Accelerated CMR using parallel imaging and prior knowledge driven methods are increasingly utilized to improve scanning efficiency and image quality of 2D and 3D applications. Many clinical 2D protocols make already use of these methods with net accelerations of up to four-fold for parallel imaging and up to five-fold for prior knowledge driven methods.

Speed-up techniques continue to develop. The wider availability of 32-channel MR systems will foster studies into the practical limits. Clearly, 3D imaging techniques will be at the focus according to theoretical predictions of noise amplification in parallel imaging. It is foreseen that the range of practically applicable reduction factors will extend to three to four in 2D imaging while large 3D volumetric imaging will be accelerated by up to factors of eight to ten [48]. Beyond those limits, the resulting signal-to-noise ratio will inherently set the bound. With the advent of many-element coil arrays careful considerations of coil calibration are required as individual coil elements are small with considerable changes in sensitivity as a function of space. It is likely that the resolution of calibration data needs to be improved leading to additional time to be spent thereby offsetting the initial speed-gain if auto-calibration is used. When acquiring separate calibration data as with SENSE implementations, the effect of spatial misregistration due to motion in-between the calibration and actual data is amplified prompting for some precautions or registration steps.

On the other side, a very practical advantage of using large coil arrays with their inherent coverage is the relative insensitivity to misplacement of the coil on the patient's chest. This simplifies patient setup and avoids coil re-adjustment for different target territories.

The field of prior knowledge driven methods currently enjoys vivid research activities. Several modifications to the original *k-t *BLAST/*k-t *SENSE scheme have been proposed addressing some of the current shortcomings [24,79]. Another very interesting and potential approach uses the Compressed Sensing framework [80,81]. In Compressed Sensing data are undersampled randomly leading to incoherent aliasing. Using iterative reconstruction methods, the aliasing can be resolved. It has been shown that such an approach is well suited for speeding up object data that are particularly sparse in the image domain itself as it is case with angiography or using a suitable sparsifying transformation [23].

Ultimately, speed-up techniques are required to provide the most appropriate balance between noise amplification and image fidelity for a given reduction factor. It is the purpose of ongoing and future research to determine the faithful range of reduction factors in combination with a suitable encoding and reconstruction strategy for all required CMR applications. It has to be a clear aim to provide unifying assessment of image quality and fidelity for all the different acceleration techniques available to allow comparisons and robust predictions.

In conclusion, parallel imaging and prior knowledge driven methods have become very valuable tools to streamline and simplify CMR exams. Their impact is expected to develop rapidly over the next years with the wider availability of multi-channel MR systems. Several methods are available which differ in their performance depending on application requiring careful considerations when setting up imaging protocols.

## Competing interests

The authors declare that they have no competing interests.
